# Genetic diversity and evolution of nine avirulence genes in South Korean *Phytophthora sojae* populations

**DOI:** 10.3389/fmicb.2026.1909451

**Published:** 2026-08-14

**Authors:** Rahel Dinsa Guta, Youngnam Yoon, Jun Hyoung Jeon, Hyeon Su Lee, Seoyeon Hong, Ok Jae Won, Rameswor Maharjan, Yi Hwi Jong, Yun-Woo Jang

**Affiliations:** 1 Smart Agricultural Technology Division, National Institute of Crop and Food Science, RDA, Miryang, Republic of Korea; 2 Rural Development Administration, Jeonju, Republic of Korea

**Keywords:** avirulence genes, genetic diversity, haplotype, pathotype, *Phytophthora sojae*, soybean root and stem rot

## Abstract

**Introduction:**

*Phytophthora sojae*, the causal agent of soybean root and stem rot, is a highly destructive pathogen affecting soybean production worldwide. Rapid evolution of avirulence (*Avr*) genes poses a major challenge to the development of durable host resistance. However, comprehensive information on the diversity, sequence variation, and evolution of multiple *Avr* genes in South Korean *P. sojae* populations remains limited. This study aimed to characterize the pathogenicity, pathotypes, and genetic diversity of nine *Avr* genes in *P. sojae* populations from the Republic of Korea.

**Methods:**

Fifty *P. sojae* isolates were collected from soybean-growing regions in the Republic of Korea between 2020 and 2025. Pathogenicity was evaluated in the soybean cultivar ‘Daewon’, and pathotypes were determined by amplification patterns of nine race-specific *Avr* genes (*Avr1a*, *Avr1b*, *Avr1c*, *Avr1d*, *Avr1k*, *Avr3a*, *Avr3b*, *Avr3c*, *Avr4/6*). Sequence polymorphism, nucleotide and amino acid variation, and haplotype diversity were analyzed to investigate the evolutionary patterns of these *Avr* genes.

**Results:**

Most isolates were highly virulent on the soybean cultivar ‘Daewon’, with 70% causing severe disease symptoms. Five distinct pathotypes were identified, with pathotype 3, characterized by the absence of *Avr3a*, representing the predominant group. Gene deletions occurred most frequently in *Avr3a* and *Avr1a*. Sequence analyses revealed extensive nucleotide and amino acid polymorphisms among *Avr* loci, particularly within the C-terminal regions of effector proteins, whereas *Avr4/6* remained highly conserved across all isolates. Haplotype analysis demonstrated substantial allelic diversity, with *Avr1a* exhibiting the highest haplotype diversity and the greatest number of haplotypes. The coexistence of multiple pathotypes and haplotypes within the same geographic regions indicated considerable genetic diversity within local *P. sojae* populations.

**Discussion:**

This study provides the first comprehensive characterization of the diversity and evolution of nine *Avr* genes in South Korean *P. sojae* populations. The results demonstrate that both gene loss and sequence diversification contribute to *Avr* gene evolution and pathogen adaptation. These findings improve our understanding of the evolutionary dynamics of *P. sojae* populations and highlight the importance of continuous pathogen surveillance to support durable resistance breeding and effective management of soybean root and stem rot.

## Introduction

Oomycetes are filamentous eukaryotic organisms that morphologically resemble fungi, but have evolved independently and are not closely related to them (Rodenburg et al., 2021). They are a diverse group of eukaryotic organisms occupying a wide range of ecological niches, with over 60% of known species being plant parasites (Rossmann et al., 2021; Thines and Kamoun, 2010). *Phytophthora*, downy mildews, and *Pythium* are well-known plant-parasitic pathogens within the class Oomycetes. They have probably evolved from a common plant-parasitic ancestor (Kamoun et al., 2015). They are among the most destructive plant pathogens and have been linked to numerous disease epidemics (Dinsa Guta et al., 2024). *Phytophthora* is considered one of the most historically important and economically impactful genera in plant pathology (Brasier et al., 2022). More than 260 *Phytophthora* species have been recognized worldwide, many of which are highly pathogenic to economically important crops (Horta Jung et al., 2024). Six out of the top 10 oomycetes belong to the genus *Phytophthora*, with *P. sojae* ranking fourth (Kamoun et al., 2015).

*Phytophthora sojae* Kaufmann and Gerdemann is a soil-borne hemibiotrophic oomycete pathogen that is homothallic, indicating the capacity for self-fertilization and production of viable oospores in the absence of a complementary genotype (McCoy et al., 2023). This pathogen can infect soybean plants at all developmental stages, from germination to maturity, and cause damage at any point during the growing season (Dussault-Benoit et al., 2020). *Phytophthora sojae* enters soybean plants via their root system, resulting in damping-off in seedlings and root rot, stem rot, and wilting in mature plants (You et al., 2024).

*Phytophthora sojae* was first observed in Indiana, USA, in 1948, and then widely distributed globally in soybean cultivation areas (Hale et al., 2023; Wu et al., 2017). The damage caused by this pathogen can reach up to 100% depending on disease severity and susceptibility of the planted cultivars (Dorrance, 2018; Hale et al., 2023). The total yield loss caused by *P. sojae* is estimated at approximately USD 1–2 billion worldwide (McCoy et al., 2023). In the Republic of Korea, soybean Phytophthora stem and root rot (PSRR) was first reported in Chungnam Province in 1996, a major soybean-producing region that accounted for approximately 15% of the country’s 156,000 metric tons of soybean production in 2025 (Heo et al., 2024; U.S. Department of Agriculture Foreign Agricultural Service (USDA-FAS), 2026). The first identified isolate of *P. sojae* (P-9664) from Chungnam has not been considered a major concern for two decades, until it emerged as a major problem in paddy fields used for soybean cultivation (Kang et al., 2019).

PSRR is effectively controlled by breeding plants with one or more dominant resistance genes called *Rps* (resistant to *P. sojae*). However, because these genes target specific *Avr* of *P. sojae*, their effectiveness is limited to only certain isolates (Jang and Lee, 2020). This concept is called gene-for-gene, in which a relationship exists between a host resistance gene and pathogen *Avr* gene. *Avr* genes in *P. sojae* encode RxLR (Arginine-any amino acid-Leucine-Arginine) effector proteins that are activated only during infection, and most of these proteins have RxLR and dEER (Aspartic acid-Glutamic acid-Glutamic acid-Arginine) motifs that are essential for their translocation into host cells (Santhanam et al., 2024). On the other hand, *Rps* genes in the host are known or expected to encode immune receptors with nucleotide-binding and leucine-rich repeat domains that detect specific pathogen effectors containing RxLR and dEER motifs. This recognition activates effector-triggered immunity in host plants (Arsenault-Labrecque et al., 2022). However, pathogens can evade detection by *Rps* genes through various mutations, including substitutions, frameshifts, partial or complete gene deletions, large insertions, recombination events, or altered expression of *Avr* genes (Arsenault-Labrecque et al., 2018). Consequently, the diversity and evolution of *Avr* genes play a critical role in determining pathogen virulence and the effectiveness of soybean resistance.

The *P. sojae* genome encodes more than 390 RxLR effectors (Madina et al., 2022), including several *Avr* genes, such as *Avr1a*, *Avr1b*, *Avr1c*, *Avr1d*, *Avr1k*, *Avr3a/5*, *Avr3b*, *Avr3c*, and *Avr4/6*, which play key roles in pathotype classification (Dorrance, 2018; Dussault-Benoit et al., 2020). Pathogenic diversity of *P. sojae* was first documented in 1955 (Zhang et al., 2010). Deployment of novel *Rps* genes in soybean cultivars drives mutation of *Avr* genes in *P. sojae*, resulting in more than 200 virulent pathotypes and diminishing long-term effectiveness of *Rps*-mediated resistance (Tremblay et al., 2021). Effective use of *Rps* genes against *P. sojae* requires matching them with *Avr* genes in local isolates, making pathotype identification through the detection of functional *Avr* genes essential (Dussault-Benoit et al., 2020). Therefore, identifying pathotypes is essential for selecting soybean varieties with specific race resistance needed for each field.

However, information regarding the diversity, sequence variation, haplotype structure, and evolutionary patterns of multiple *Avr* genes in South Korean *P. sojae* populations remains limited. Therefore, in this study, the *Avr* genes of *P. sojae* isolates were investigated to assess the genetic and pathogenic diversity of the pathogen population. A total of fifty isolates were analyzed using nine race-specific *P. sojae Avr* gene primers to identify pathotypes based on the presence or absence of specific *Avr* genes. Successfully amplified isolates were further subjected to sequence polymorphism and haplotype analyses to evaluate genetic variation among the examined loci. Through comparative sequence analysis, the study examined *Avr* gene distribution patterns, genetic variation, evolutionary differentiation, and potential pathogenic diversity within regional *P. sojae* populations. These findings provide baseline information for future disease monitoring, soybean resistance breeding, and effective disease-management strategies.

## Materials and methods

### Sample collection

Symptomatic soybean plants were collected from 12 soybean-growing regions in South Korea between 2020 and 2025, including South Gyeongsang Province (Miryang and Jinju), North Gyeongsang Province (Sangju and Andong), Gyeongsang Province (Daegu), South Jeolla Province (Jangseong and Naju), and North Jeolla Province (Buan, Gimje, Gochang, Sunchang, and Wanju).

### Pathogen isolation

*P. sojae* isolates were cultured on 10% V8–CaCO_3_ agar medium. To prepare the growth medium, 100 mL of V8 juice was added to 900 mL of distilled water. To this mixture, 1.0 g CaCO_3_ and 17 g Bacto agar were added, and the solution was thoroughly mixed using a magnetic stirrer and autoclaved at 121 °C for 15 min. The mixture was allowed to cool to approximately 50 °C and mixed again with a magnetic stirrer to ensure complete dissolution of CaCO_3_. The medium was then dispensed into petri dishes.

Infected soybean stems were cut into pieces and surface-sterilized with 0.5% sodium hypochlorite solution. Subsequently, five stem pieces were placed on 10% V8–CaCO_3_ agar media and incubated at 25 °C for 12 days.

### Morphological and molecular identification

Morphological characterization of the *P. sojae* isolates was performed using 7-day-old cultures. Sexual and asexual reproductive structures, including oogonia, oospores, and sporangia, were examined under an Axio Imager A2 compound microscope equipped with an Axiocam 305 color camera (Carl Zeiss, Oberkochen, Germany) at magnifications of 20× −40×. The morphology of each structure, including its shape, size, and other diagnostic characteristics, was assessed to facilitate species identification and comparison among isolates.

For molecular identification, approximately 25 mg of fresh mycelium from each isolate was harvested by dislodging with sterilized distilled water, centrifuged at 4,000 rpm for 15 min, and used for genomic DNA extraction. DNA was extracted using i-genomic BYF DNA extraction kit, following the manufacturer’s instructions. The internal transcribed spacer (ITS) region of *P. sojae* isolates was amplified using the primer pairs ITS1 (5′-TCCGTAGGTGAACCTGCGG -3′) and ITS4 (5′-TCCTCCGCTTATTGATATGC -3′). All generated sequences have been deposited in the GenBank database, and the accession numbers are provided in Supplementary Table S1.

### Pathogenicity test

Seven-day-old “Daewon”, a PSRR-resistant soybean variety (Jang et al., 2020), was inoculated using the hypocotyl split inoculation method. Specifically, 10 soybean seedlings for each *P. sojae* isolates were planted, and 1 week after planting, a 1-cm slit was generated at the center of the hypocotyl. Inoculum preparation and hypocotyl inoculation were performed according to the method described by Kang et al. (2019). *Phytophthora sojae* isolates were cultured on 10% V8–CaCO_3_ agar media for 7 days. The colonized agar was transferred into a 50-mL syringe and homogenized by passing it through the syringe twice immediately before inoculation to produce a mycelial slurry. Subsequently, 0.3 mL of mycelial slurry from each isolate was injected into the slit using a 10-mL syringe with an 18-gauge needle. Inoculated seedlings were incubated in a controlled chamber at 25 °C with 80% humidity and a 12-h light/12-h dark photoperiod for 5 days. At 5 days of inoculation (DAI), their genotypic reactions were determined as previously described (Heo et al., 2024): resistant (R) if <30% dead seedlings, susceptible (S) if >70% dead seedlings, or intermediate (I) if 30%–70% dead seedlings. The experiment was conducted thrice with three biological replicates.

### Pathotype characterization

Specific primers for nine *P. sojae Avr* genes were designed based on previously published full-length gene sequences, with additional flanking sequences included to ensure complete amplification of the target regions (Table 1). Isolates were amplified using these primers to characterize the pathotypes. The polymerase chain reaction (PCR) conditions were as follows: an initial denaturation at 94 °C for 5 min, followed by 30 cycles of denaturation at 94 °C for 30 s, annealing at 57–65 °C for 30 s, extension at 72 °C for 1 min, and final extension at 72 °C for 5 min.

**Table 1:** Full-length primers used in this study and their description.

Primers	Forward sequence	Reverse sequence	Product size (bp)
Avr1a	ACAATGCGCCTAACCAACACCCTCG	TATCTAATGACCTCTCAAGTGGAAT	381
Avr1b	ACCATGCGTCTATCTTTTGTGCTTT	TTCTCAGCTCTGATACAGGTGAAAG	417
Avr1c	ACAATGCGCCTAACCAACACCCTCG	TATCTAATGACCTCTCAAGTGGAAT	381
Avr1d	GCTATGCGTCTCTCCAGCACCACCT	GAACTAATTCATGCGGTAGTGGATT	378
Avr1 k	ATTATGTTTCTTCTTCAGCGTGCGT	AGCTCAGATAATCATGATGCTGTTC	747
Avr3a	AAGATGCGCCTCGCTCAAGTTGTGG	CGGCTACGCGTTTTTCGCTGCTGCC	330
Avr3b	AATATGCGCTTCCTGTTCCTGTTGG	ACGTTACCGTACCCCTCCTTCTTCC	684
Avr3c	AAGATGCGCGTGTGCTCCGTCCTCC	AAGTTACTTGTGTTTCCTTCGGTAT	663
Avr4/6	ATGATGGGCCTCCACAAGGGCTTCT	AACTTACGTTAGGTGGTGTAGTCCG	372

### DNA sequence polymorphism and haplotype analysis

The purified PCR products were sequenced using the Sanger dideoxy method. Forward and reverse sequences were assembled, aligned, and trimmed using BioEdit, with manual corrections applied where necessary. Sequence identities were initially verified through BLASTn searches against the NCBI database. Multiple sequence alignments of nucleotide and deduced amino acid sequences were performed using ClustalW and MEGA v.11. Nucleotide sequences were translated into amino acid sequences using the Expasy translate tool. All nucleotide and amino acid sequences generated in this study were deposited in the GenBank database.

Sequence polymorphism and haplotype analyses were conducted using DnaSP v.6.12.03, following sequence alignment. The number of polymorphic sites (*Ps*), haplotypes (*Hn*), haplotype diversity (*Hd*), nucleotide diversity (*π*), and average number of pairwise nucleotide differences (K) were calculated to quantify sequence variation among *P. sojae* isolates. Neutrality was assessed using Tajima’s D and Fu and Li’s D and F statistics. GeneDoc was used to visualize the single-nucleotide polymorphisms (SNPs) among isolates. Pairwise sequence identity among *P. sojae* isolates was analyzed using Sequence Demarcation Tool v.1.3, to determine the degree of nucleotide sequence similarity among isolates for each *Avr* gene. A heatmap was constructed using MEGA11 and TBtools.

## Results

### Characteristics of *Phytophthora sojae* isolates

**Figure 1:**
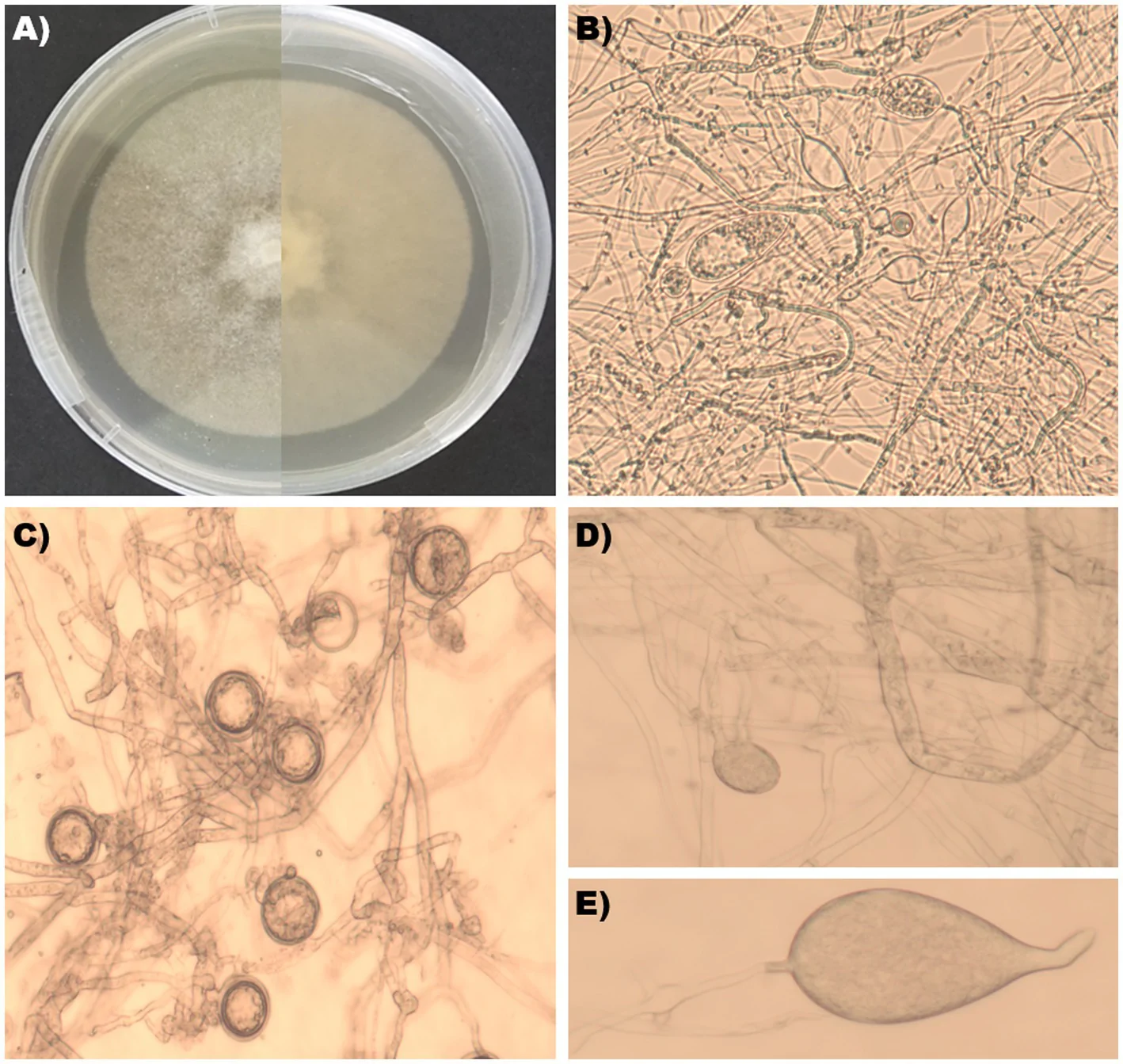
Morphological characteristics of *P. sojae*. **(A)** Front and reverse views of *P. sojae* colonies grown on 10% V8–CaCO_3_ agar medium for 7 days; **(B)** Coenocytic hyphae with numerous developing oogonia produced in culture; **(C)** Mature thick-walled oospores formed within oogonia; **(D)** Immature oogonium; and **(E)** Papillate sporangium. Images **(B–E)** were captured using a compound microscope (scale bar = 20 μm).

A total of 50 isolates were identified as *P. sojae* based on morphological characteristics and molecular analyses (Figure 1; Supplementary Table S1). On 10% V8–CaCO_3_ agar medium, the isolates produced well-developed, white, fluffy mycelial colonies. Microscopic examination revealed thick, hyaline, aseptate hyphae with abundant sexual structures. The oogonia were thin-walled, spherical structures borne on stalks, while the mature oospores were thick-walled and spherical. The sporangia were papillate and lemon-shaped (Figure 1).

### Disease severity of soybean induced by *Phytophthora sojae* isolates

**Figure 2:**
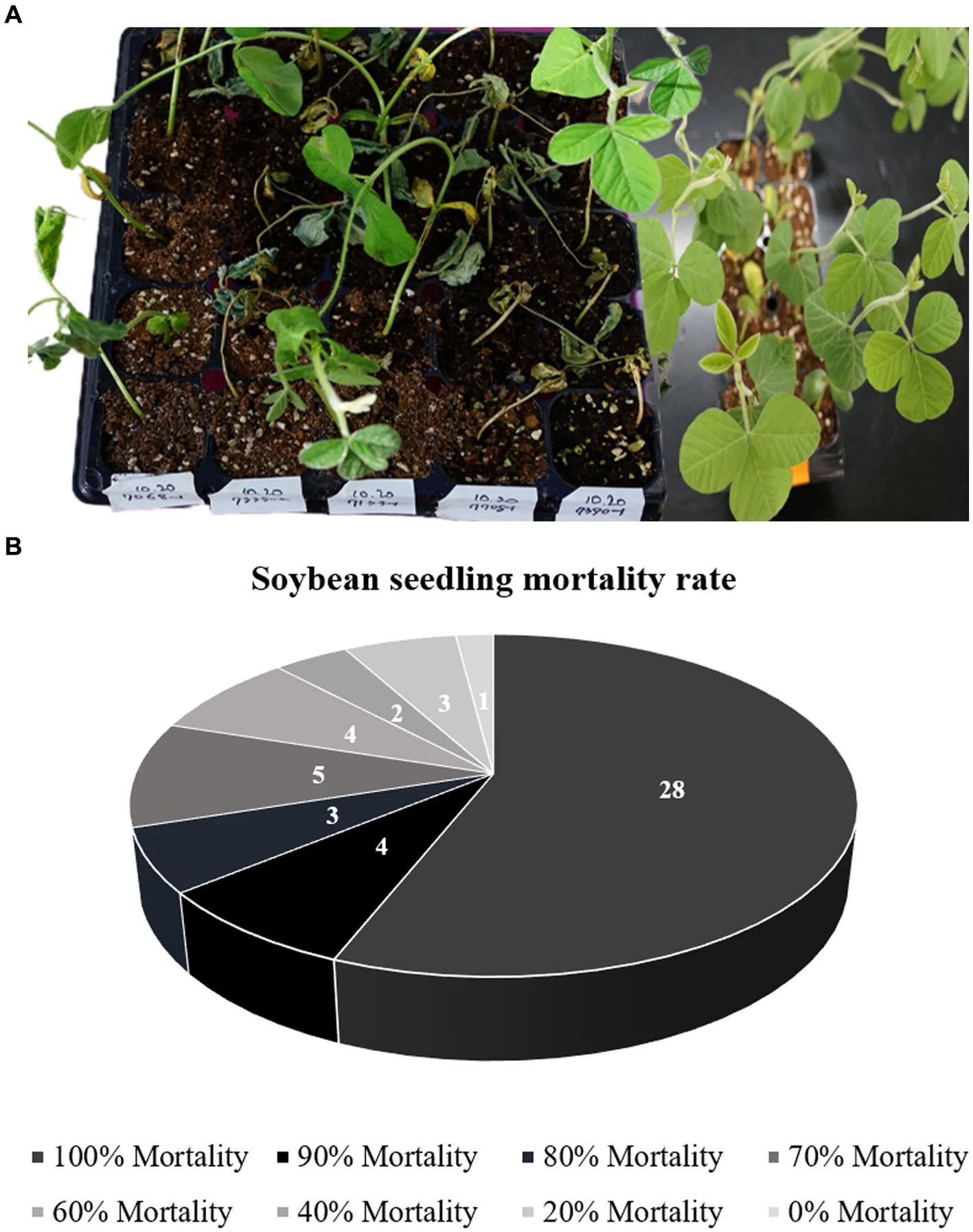
Pathogenicity assay of *P. sojae* isolates from the Republic of Korea. **(A)** Soybean seedlings inoculated with different *P. sojae* isolates at 5 days after inoculation, with control plants shown on the right; **(B)** Percent mortality caused by *P. sojae* isolates.

Pathogenicity assay revealed that most *P. sojae* isolates were highly virulent on “Daewon”. Disease severity assessed at 5 DAI showed that 35 of the 50 isolates (70%) caused severe disease symptoms on the soybean cultivar carrying the *Rps1* and *Rps7* resistance genes (Figure 2).

In contrast, isolate MR_5522–1 produced no visible disease symptoms, with all 10 inoculated seedlings remaining healthy and alive 5 DAI. Consequently, this isolate exhibited the lowest disease severity level (0%), whereas isolates DG_3968–2, GC_4351–1, and JS_5823–1 were resistant to soybean seedlings, each showing 20% disease severity. The remaining isolates exhibited intermediate levels of disease severity (Table 2).

**Table 2:** Pathotype classification of 50 *P. sojae* isolates with their phenotypic reaction.

Pathotypes	*Avr* factors	Isolates	Percent disease severity	Disease reaction on Daewon cultivar
Pathotype 1	0	DG_3191	70	I
		WJ_3352	100	S
		AD_3617	90	S
		WJ_3624	90	S
		WJ_3849	70	I
		AD_3850	100	S
		SC_4025–4	100	S
		SJ_4286	70	I
		AD_4379	60	I
		SC_4536–3	100	S
		WJ_4609	80	S
		SC_4816–2	100	S
		DG_5097	70	I
		GJ_5219	100	S
		SJ_7003–1	100	S
Pathotype 2	1a	AD_3035	100	S
		JJ_5053–1	100	S
Pathotype 3	3a	GJ_3053	90	S
		GJ_3170	80	S
		AD_3213–1	60	I
		DG_3229	80	S
		GJ_3233	100	S
		GJ_3385	40	I
		GJ_3541	100	S
		SJ_3613	70	S
		WJ_3615	100	S
		GJ_3856	90	S
		DG_3968–2	20	R
		GC_4351–1	20	R
		MR_4775–1	100	S
		GC_4806	100	S
		GJ_4920	100	S
		SJ_4935	100	S
		JS_5007–1	40	I
		GJ_5052–1	100	S
		NJ_5296–1	100	S
		GJ_5450–1	100	S
		MR_5503–1	100	S
		MR_5522–1	0	R
		GJ_5575–1	100	S
		JS_5823–1	20	R
		DG5997-2	100	S
		AD_7656–1	100	S
		GJ_7896–1	60	I
Pathotype 4	1a, 3a	MR_5148–1	60	I
		MR_5252–1	100	S
		MR_5420	100	S
		BA_5550–5	100	S
Pathotype 5	1b, 3a	MR_4979–1	100	S
		GJ_4986	100	S

S, susceptible; R, resistant; I, intermediate.

### Distribution of *Avr* genes and pathotype diversity of *Phytophthora sojae* isolates

**Figure 3:**
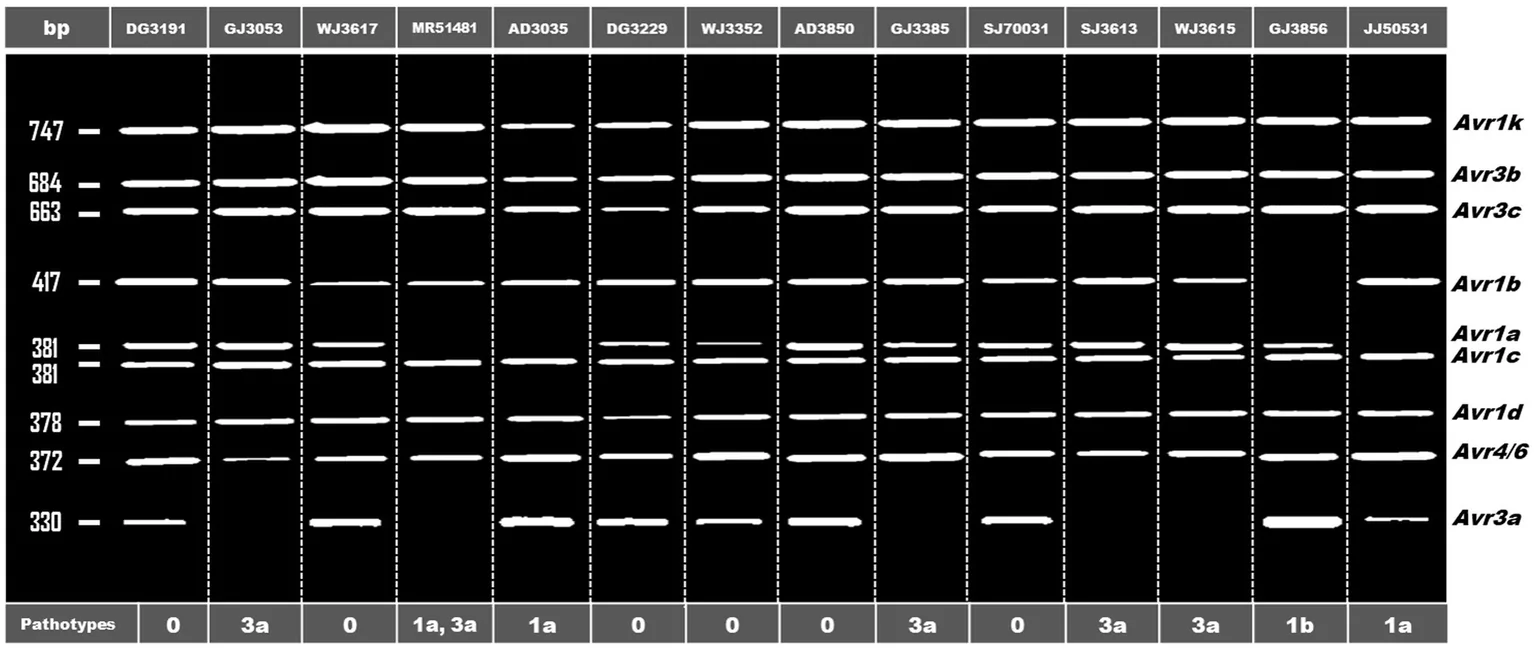
Polymerase chain reaction (PCR) amplification profiles of nine *P. sojae* *Avr* genes in selected isolates. Isolates were amplified using gene-specific primer sets targeting *Avr1a*, *Avr1b*, *Avr1c*, *Avr1d*, *Avr1 k*, *Avr3a*, *Avr3b*, *Avr3c*, and *Avr4/6*. The expected amplicon sizes (bp) for each *Avr* gene are indicated on the left. Pathotypes, determined based on the presence or absence of *Avr* gene amplification, are shown below each isolate.

PCR amplification identified the presence or absence of specific *Avr* genes within each isolate (Figure 3). *Avr3a* exhibited the highest deletion frequency, with 33 of 50 isolates lacking the corresponding locus. Deletions of *Avr1a* and *Avr1b* were detected in six and two isolates, respectively. In contrast, *Avr1c*, *Avr1d*, *Avr1k*, *Avr3b*, *Avr3c*, and *Avr4/6* were successfully amplified in all isolates.

Based on the presence–absence patterns of *Avr* genes, the isolates were classified into five distinct pathotypes. Pathotype 3, characterized by the absence of the *Avr3a* locus, was the most prevalent group, comprising 54% of the isolates. In contrast, pathotypes 2 and 5 were least common, each representing only 4% of the population (Table 2).

Notably, isolates originating from the same geographical region were often assigned to different pathotypes, indicating substantial pathotype diversity within local populations. For example, isolates collected from Miryang lacked at least one *Avr* gene in their genomes and were therefore classified into three pathotype classes. Similarly, isolates collected from Andong and Gimje were classified into three distinct pathotypes, despite sharing the same geographical origin.

Additionally, isolates belonging to the same pathotype group exhibited varying levels of pathogenicity. Pathotype 3, the most predominant pathotype, included isolates that showed resistant, intermediate, and susceptible characteristics on the soybean cultivar. Despite sharing the same presence–absence profile of *Avr* genes, sequence variation within these genes, including nucleotide substitutions causing amino-acid changes, may have contributed to the observed differences in pathogenicity.

### Sequence polymorphism of *Avr* genes in *Phytophthora sojae* isolates

**Figure 4:**
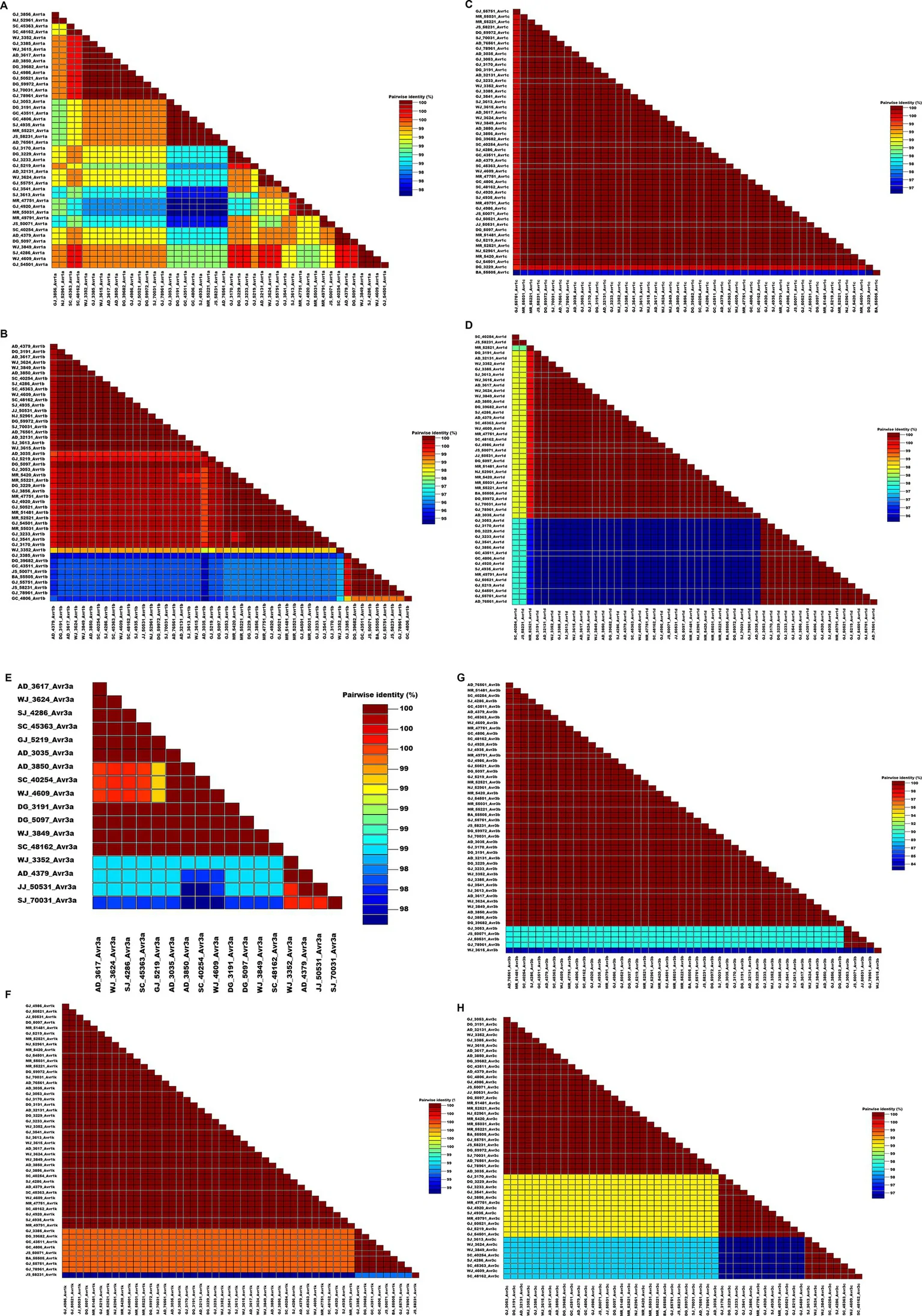
Pairwise nucleotide sequence identity matrices of nine *Avr* genes in the South Korean *P. sojae* population. Pairwise nucleotide sequence identities among 50 *P. sojae* isolates calculated from aligned sequences of **(A)** *Avr1a*, **(B)** *Avr1b*, **(C)** *Avr1c*, **(D)** *Avr1d*, **(E)** *Avr1 k*, **(F)** *Avr3a*, **(G)** *Avr3b*, and **(H)** *Avr3c* using Sequence Demarcation Tool (SDT). Rows and columns correspond to individual isolates. Cell colors represent pairwise sequence identity values, with cooler colors indicating greater sequence similarity and warmer colors indicating higher sequence similarity.

Comparative analysis of nucleotide and amino acid sequences of nine *Avr* loci were performed using 50 *P. sojae* isolates. The resulting sequences’ GenBank accession numbers are provided in Table 3. The result of these analyses revealed no insertion–deletion and variation in sequence length among isolates; however, SNPs were detected. Pairwise sequence identities among isolates ranged from 84% to 100% across the nine *Avr* genes, with *Avr3b* exhibiting the lowest sequence identity (Figure 4). *Avr3b* exhibited the highest genetic diversity, with 397 polymorphic sites. In contrast, *Avr4/6* was highly conserved, with no detectable nucleotide or amino acid variations across all isolates (Table 4).

**Table 3:** Description of 50 *P. sojae* isolates used in this study and GenBank accession numbers of their nine *Avr* gene sequences.

No.	Date	Isolates	Location	GenBank accession numbers								
				*1a*	*1b*	*1c*	*1d*	*1k*	*3a*	*3b*	*3c*	*4/6*
1	2020-06-23	AD_3035	Andong	—	PZ311468	PZ311520	PZ337312	PZ337362	PZ337265	PZ337412	PZ337462	PZ337512
2	2019-08-06	GJ_3053	Gimje	PZ441120	PZ311469	PZ311521	PZ337313	PZ337363	—	PZ337413	PZ337463	PZ337513
3	2019-07-03	GJ_3170	Gimje	PZ441121	PZ311470	PZ311522	PZ337314	PZ337364	—	PZ337414	PZ337464	PZ337514
4	2019-07-09	DG_3191	Daegu	PZ441122	PZ311471	PZ311523	PZ337315	PZ337365	PZ337266	PZ337415	PZ337465	PZ337515
5	2020-06-23	AD_3213–1	Andong	PZ441123	PZ311472	PZ311524	PZ337316	PZ337366	—	PZ337416	PZ337466	PZ337516
6	2019-07-03	DG_3229	Daegu	PZ441124	PZ311473	PZ311525	PZ337317	PZ337367	—	PZ337417	PZ337467	PZ337517
7	2019-07-03	GJ_3233	Gimje	PZ441125	PZ311474	PZ311526	PZ337318	PZ337368	—	PZ337418	PZ337468	PZ337518
8	2019-07-02	WJ_3352	Wanju	PZ441126	PZ311475	PZ311527	PZ337319	PZ337369	PZ337267	PZ337419	PZ337469	PZ337519
9	2019-07-03	GJ_3385	Gimje	PZ441127	PZ311476	PZ311528	PZ337320	PZ337370	—	PZ337420	PZ337470	PZ337520
10	2019-07-03	GJ_3541	Gimje	PZ441128	PZ311477	PZ311529	PZ337321	PZ337371	—	PZ337421	PZ337471	PZ337521
11	2019-07-03	SJ_3613	Sangju	PZ441129	PZ311478	PZ311530	PZ337322	PZ337372	—	PZ337422	PZ337472	PZ337522
12	2019-07-02	WJ_3615	Wanju	PZ441130	PZ311479	PZ311531	PZ337323	PZ337373	—	PZ337423	PZ337473	PZ337523
13	2019-07-04	AD_3617	Andong	PZ441131	PZ311480	PZ311532	PZ337324	PZ337374	PZ337268	PZ337424	PZ337474	PZ337524
14	2019-07-03	WJ_3624	Wanju	PZ441132	PZ311481	PZ311533	PZ337325	PZ337375	PZ337269	PZ337425	PZ337475	PZ337525
15	2019-07-02	WJ_3849	Wanju	PZ441133	PZ311482	PZ311534	PZ337326	PZ337376	PZ337270	PZ337426	PZ337476	PZ337526
16	2019-07-04	AD_3850	Andong	PZ441134	PZ311483	PZ311535	PZ337327	PZ337377	PZ337271	PZ337427	PZ337477	PZ337527
17	2019-07-03	GJ_3856	Gimje	PZ441135	PZ311484	PZ311536	PZ337328	PZ337378	—	PZ337428	PZ337478	PZ337528
18	2020-06-26	DG_3968–2	Daegu	PZ441136	PZ311485	PZ311537	PZ337329	PZ337379	—	PZ337429	PZ337479	PZ337529
19	2024-08-23	SC_4025–4	Sunchang	PZ441137	PZ311486	PZ311538	PZ337330	PZ337380	PZ337272	PZ337430	PZ337480	PZ337530
20	2019-06-16	SJ_4286	Sangju	PZ441138	PZ311487	PZ311539	PZ337331	PZ337381	PZ337273	PZ337431	PZ337481	PZ337531
21	2019-07-17	GC_4351–1	Gochang	PZ441139	PZ311488	PZ311540	PZ337332	PZ337382	—	PZ337432	PZ337482	PZ337532
22	2019-07-04	AD_4379	Andong	PZ441140	PZ311489	PZ311541	PZ337333	PZ337383	PZ337274	PZ337433	PZ337483	PZ337533
23	2024-08-23	SC_4536–3	Sunchang	PZ441141	PZ311490	PZ311542	PZ337334	PZ337384	PZ337275	PZ337434	PZ337484	PZ337534
24	2019-07-03	WJ_4609	Wanju	PZ441142	PZ311491	PZ311543	PZ337335	PZ337385	PZ337276	PZ337435	PZ337485	PZ337535
25	2025-06-19	MR_4775–1	Miryang	PZ441143	PZ311492	PZ311544	PZ337336	PZ337386	—	PZ337436	PZ337486	PZ337536
26	2019-07-17	GC_4806	Gochang	PZ441144	PZ311493	PZ311545	PZ337337	PZ337387	—	PZ337437	PZ337487	PZ337537
27	2024-08-23	SC_4816–2	Sunchang	PZ441145	PZ311494	PZ311546	PZ337338	PZ337388	PZ337277	PZ337438	PZ337488	PZ337538
28	2019-07-03	GJ_4920	Gimje	PZ441146	PZ311495	PZ311547	PZ337339	PZ337389	—	PZ337439	PZ337489	PZ337539
29	2019-06-14	SJ_4935	Sangju	PZ441147	PZ311496	PZ311548	PZ337340	PZ337390	—	PZ337440	PZ337490	PZ337540
30	2025-06-19	MR_4979–1	Miryang	PZ441148	—	PZ311549	PZ337341	PZ337391	—	PZ337441	PZ337491	PZ337541
31	2025-06-25	GJ_4986	Gimje	PZ441149	—	PZ311550	PZ337342	PZ337392	—	PZ337442	PZ337492	PZ337542
32	2024-07-08	JS_5007–1	Jangseong	PZ441150	PZ311497	PZ311551	PZ337343	PZ337393	—	PZ337443	PZ337493	PZ337543
33	2024-07-22	GJ_5052–1	Gimje	PZ441151	PZ311498	PZ311552	PZ337344	PZ337394	—	PZ337444	PZ337494	PZ337544
34	2024-07-18	JJ_5053–1	Jinju	—	PZ311499	PZ311553	PZ337345	PZ337395	PZ337278	PZ337445	PZ337495	PZ337545
35	2021-06-29	DG_5097	Daegu	PZ441152	PZ311500	PZ311554	PZ337346	PZ337396	PZ337279	PZ337446	PZ337496	PZ337546
36	2024-07-01	MR_5148–1	Miryang	—	PZ311501	PZ311555	PZ337347	PZ337397	—	PZ337447	PZ337497	PZ337547
37	2024-07-08	GJ_5219	Gimje	PZ441153	PZ311502	PZ311556	PZ337348	PZ337398	PZ337280	PZ337448	PZ337498	PZ337548
38	2024-07-01	MR_5252–1	Miryang	—	PZ311503	PZ311557	PZ337349	PZ337399	—	PZ337449	PZ337499	PZ337549
39	2024-07-18	NJ_5296–1	Naju	PZ441154	PZ311504	PZ311558	PZ337350	PZ337400	—	PZ337450	PZ337500	PZ337550
40	2024-07-01	MR_5420	Miryang	—	PZ311505	PZ311559	PZ337351	PZ337401	—	PZ337451	PZ337501	PZ337551
41	2024-07-22	GJ_5450–1	Gimje	PZ441155	PZ311506	PZ311560	PZ337352	PZ337402	—	PZ337452	PZ337502	PZ337552
42	2024-07-01	MR_5503–1	Miryang	PZ441156	PZ311507	PZ311561	PZ337353	PZ337403	—	PZ337453	PZ337503	PZ337553
43	2024-07-12	MR_5522–1	Miryang	PZ441157	PZ311508	PZ311562	PZ337354	PZ337404	—	PZ337454	PZ337504	PZ337554
44	2023-07-03	BA_5550–5	Buan	-	PZ311509	PZ311563	PZ337355	PZ337405	—	PZ337455	PZ337505	PZ337555
45	2024-07-08	GJ_5575–1	Gimje	PZ441158	PZ311510	PZ311564	PZ337356	PZ337406	—	PZ337456	PZ337506	PZ337556
46	2024-07-08	JS_5823–1	Jangseong	PZ441159	PZ311511	PZ311565	PZ337357	PZ337407	—	PZ337457	PZ337507	PZ337557
47	2024-07-05	DG_5997–2	Daegu	PZ441160	PZ311512	PZ311566	PZ337358	PZ337408	—	PZ337458	PZ337508	PZ337558
48	2024-07-23	SJ_7003–1	Sangju	PZ441161	PZ311513	PZ311567	PZ337359	PZ337409	PZ337281	PZ337459	PZ337509	PZ337559
49	2024-09-06	AD_7656–1	Andong	PZ441162	PZ311514	PZ311568	PZ337360	PZ337410	—	PZ337460	PZ337510	PZ337560
50	2024-09-05	GJ_7896–1	Gimje	PZ441163	PZ311515	PZ311569	PZ337361	PZ337411	—	PZ337461	PZ337511	PZ337561

**Table 4:** Summary of haplotype and polymorphism analysis of *P. sojae* isolates for each *Avr* gene.

Genes	*n*	*H_n_*	*H_d_*	*P_s_*	*π*	*p*-value
*Avr1a*	44	13	0.890 ± 0.027	9	0.008	*p* > 0.10^ns^
*Avr1b*	48	8	0.707 ± 0.04	24	0.01310	*P* > 0.10^ns^
*Avr1c*	50	4	0.118 ± 0.062	63	0.00661	*p* < 0.001***
*Avr1d*	50	4	0.522 ± 0.052	17	0.02053	*P* < 0.001***
*Avr1 k*	50	3	0.308 ± 0.074	9	0.00118	*P* > 0.10^ns^
*Avr3a*	17	2	0.382 ± 0.113	4	0.00422	*P* > 0.10^ns^
*Avr3b*	50	5	0.263 ± 0.08	397	0.08729	*P* > 0.10^ns^
*Avr3c*	50	3	0.568 ± 0.056	19	0.00919	*P* > 0.10^ns^
*Avr4/6*	50	1	0.000	0	0.00000	—

***p < 0.001; ns, not significant.

Nonsynonymous mutations within the N-terminal regions of *Avr* gene were relatively uncommon. Such mutations were detected at the *Avr1a* locus in 10 isolates and at the *Avr1c* and *Avr1k* loci in one isolate each. In contrast, most nucleotide substitutions and amino acid changes were concentrated within the C-terminal regions of *Avr* genes.

### Haplotype diversity of nine *Avr* loci in *Phytophthora sojae*

**Figure 5:**
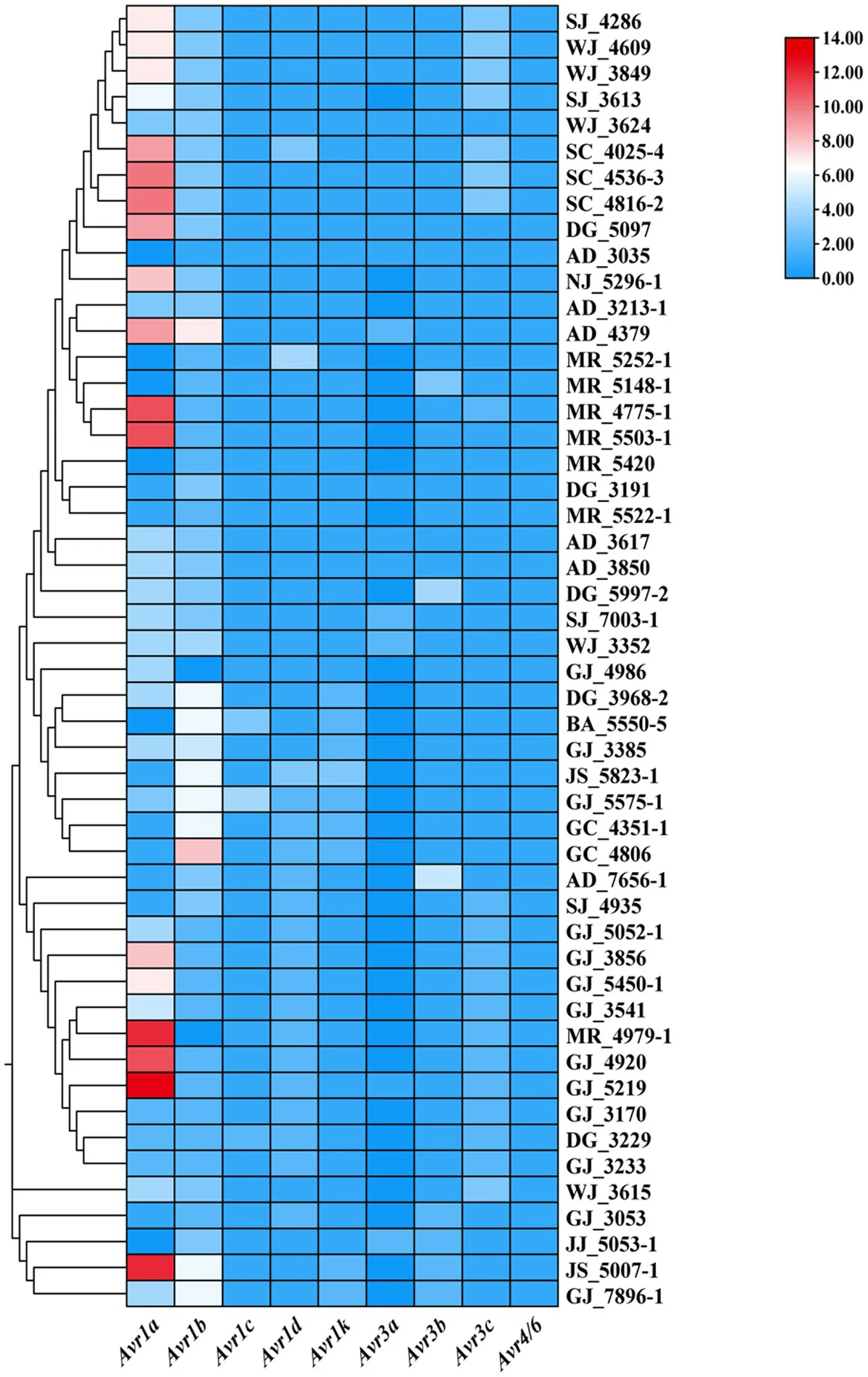
Multilocus *Avr* gene haplotype profiles of *P. sojae* isolates from the Republic of Korea. Heatmap showing haplotypes identified for nine *Avr* genes across 50 *P. sojae* isolates. Rows represent individual isolates and columns represent *Avr* genes. Distinct colors indicate different haplotypes identified within each gene. The phylogenetic tree on the left was inferred from concatenated nucleotide sequences of the nine *Avr* genes using the maximum-likelihood method. Isolates were clustered according to multilocus genetic similarity.

**Figure 6:**
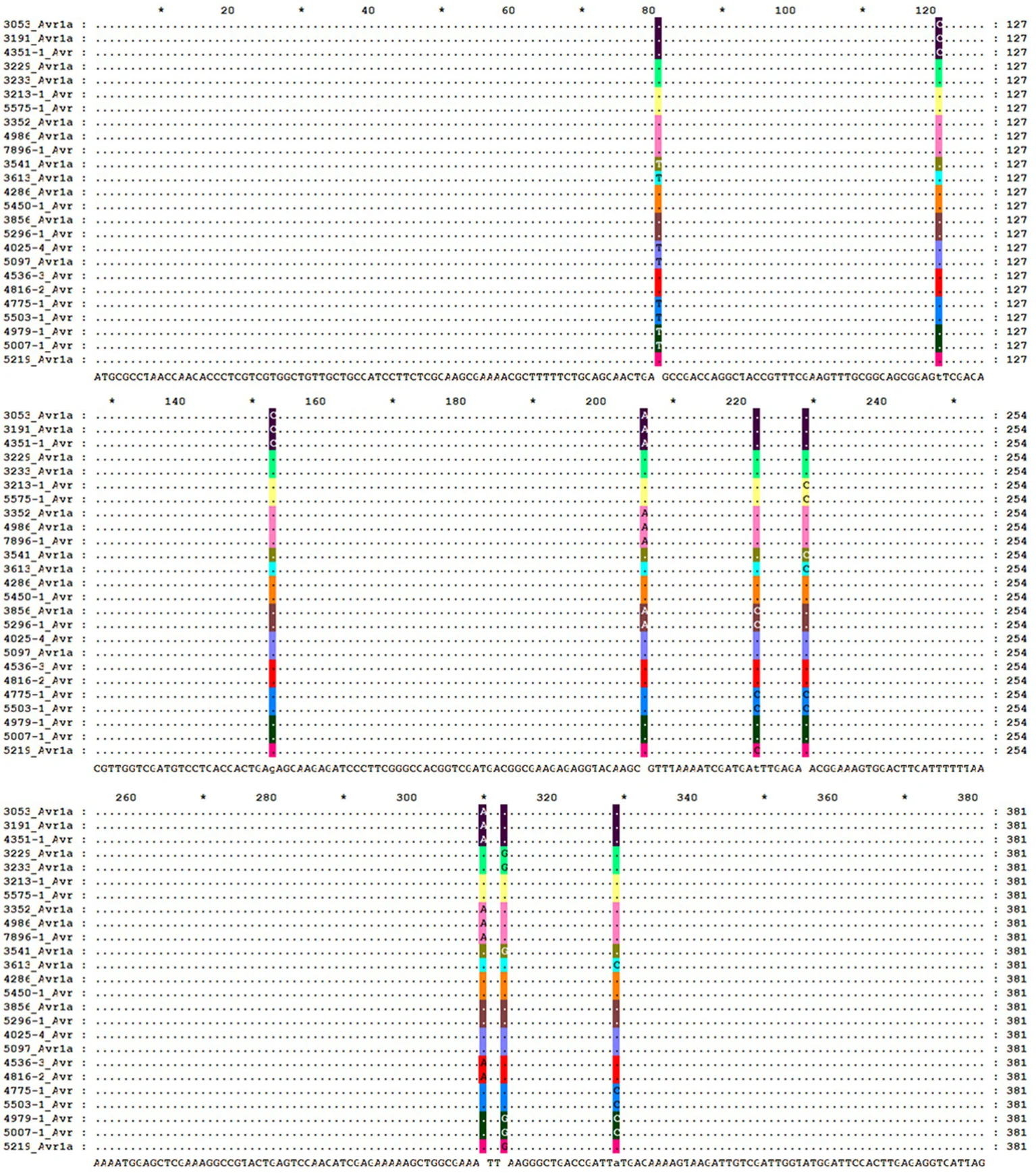
Multiple sequence alignment of *Avr1a* showing nucleotide polymorphisms. Each color shade represents 13 haplotypes in *Avr1a*.

Haplotype analysis identified 45 unique haplotypes across nine *Avr* loci (Figure 5). *Avr1a* exhibited the highest haplotype diversity, with an *Hd* of 0.890 ± 0.027 and 13 distinct haplotypes (Figure 6). The high level of diversity observed at this locus was primarily associated with nonsynonymous substitutions within the effector domain.

Across all nine *Avr* loci, the mean nucleotide diversity of the population was 0.0167, and the average *Hd* value per locus reached 0.417 ± 0.056. Although *Avr1a* showed the highest haplotype diversity, *Avr3a* contributed the largest proportion of overall sequence variation within the population, further supporting its status as a highly genetically variable *Avr* gene in *P. sojae*.

Coexistence of multiple haplotypes within isolates originating from the same geographical region was frequently observed. Notably, isolates collected from Gimje and Daegu exhibited relatively high haplotype diversity across several *Avr* loci (Supplementary Table S2).

## Discussion

*Phytophthora sojae* is the second most economically important agent causing soybean disease worldwide after soybean cyst nematodes (Chandra et al., 2022). The rapid evolution of *Avr* genes in this pathogen complicates the development of soybean cultivars with long-term effective resistance against PSRR. Sequence diversification and loss of *Avr* genes have been identified as the major mechanisms underlying *Avr* evolution in the *P. sojae* population (Zhang et al., 2019). In the present study, considerable pathogenic and genetic diversity was detected among *P. sojae* isolates collected from soybean-growing regions in the Republic of Korea. Most isolates caused severe disease symptoms and complete seedling mortality, indicating that highly virulent *P. sojae* populations are widely distributed throughout the country and may have adapted to soybean cultivars commonly cultivated in these regions.

Variation in *Avr* gene amplification patterns among isolates revealed considerable genetic diversity within the South Korean population. Although several *Avr* genes were consistently detected across all isolates, deletions of *Avr1a*, *Avr1b*, and particularly *Avr3a* were observed in multiple isolates and associated with differences in pathogenicity on “Daewon”. *Phytophthora sojae* can evade recognition by *Rps* genes through various mechanisms, including promoter disruption, sequence polymorphism, gene deletion, frameshift mutations, transcriptional variation, and cooperative interactions among effectors (Arsenault-Labrecque et al., 2022; Hou et al., 2022). Therefore, the genetic variation observed among isolates and *Avr* loci probably reflects differences in their ability to interact with and overcome host resistance genes.

The predominance of pathotype 3, characterized by the absence of *Avr3a*, suggests that selection pressure may favor isolates capable of escaping recognition by the corresponding soybean resistance gene. Similarly, deletion of *Avr1a* was associated with altered virulence phenotypes among South Korean isolates. Consistent with the findings of previous reports, structural variation contributes to the evolution of *Avr* genes in *P. sojae* (Huang et al., 2019). Although the deletion frequencies of *Avr1a* and *Avr3a* were relatively very high in the present study, deletion of *Avr1a* copies has been reported in virulent isolates, whereas *Avr3a* virulence has been associated with reduction of copy number, deletion of tandem repeat units containing *Avr3a*, and transcriptional polymorphism (Arsenault-Labrecque et al., 2022; Qutob et al., 2009). In addition, Dong et al. (2011) demonstrated that *Avr3a* is a highly polymorphic RxLR effector and that loss of *Avr3a* expression or structural variation at the *Avr3a/5* locus enables *P. sojae* to overcome soybean resistance mediated by *Rps3a* and *Rps5*. Furthermore, Qutob et al. (2013) reported that isolate ACR10 acquired virulence through small RNA-mediated epigenetic silencing of *Avr3a*, allowing the pathogen to evade recognition by the soybean resistance gene *Rps3a*.

In addition to gene deletions, extensive sequence variation was detected among the amplified *Avr* genes, indicating ongoing diversification of effector loci within the population (Figure 4). Numerous SNPs were identified across several *Avr* genes, highlighting the dynamic nature of pathogen evolution. These findings are consistent with those previously reported (Luong et al., 2025), in which substantial sequence polymorphism and genetic diversity have been observed among South Korean *P. sojae* isolates, suggesting that local populations are subject to continuous selection pressure. Similarly, increasing pathotype diversity and sequence polymorphism have been reported in *P. sojae* populations from the USA, Canada, and China between 1990 and 2019, as reflected by relatively high Gleason diversity index values (McCoy et al., 2023). Increased genetic diversity may enhance the capacity of pathogen populations to adapt to host resistance genes, thereby promoting substantial variation in virulence phenotypes. Furthermore, sequence polymorphisms within *Avr* genes can modify effector recognition by host immune receptors, thereby facilitating the emergence of virulent isolates capable of overcoming host resistance. These findings support the hypothesis that both sequence diversification and gene loss contribute to the evolution of *Avr* genes in *P. sojae*.

Our findings further revealed that sequence polymorphisms were predominantly concentrated within the C-terminus of *Avr* genes. Because the C-terminal domain of oomycete effectors is generally responsible for effector activity and interaction with host targets, variation in this region may alter effector function and host recognition (Arif et al., 2018). This is consistent with a previous finding that the RxLR effector *Avh92* of *P. sojae* was polymorphic at the C-terminal region among the isolates, resulting in the identification of three distinct *Avh92* alleles (Qutob et al., 2009). Similarly, 21 amino acid mutations in the C-terminus of *PsAvr1b* have been verified in the virulent strain P7076 (Hou et al., 2022). Yang et al. (2017) have detected 26 polymorphic amino acid sites in the C-terminal region of the *P. sojae* effector *Avh238*.

Haplotype analysis further demonstrated high genetic diversity among isolates (Figure 5). Among the nine *Avr* loci examined, *Avr1a* exhibited the highest number of haplotypes and elevated haplotype diversity, suggesting that this locus might be under relatively strong evolutionary selection pressure (Figure 6). Similar findings have been reported through haplotype analysis, revealing extensive sequence diversification among *P. sojae Avr* genes, with specific haplotypes strongly associated with virulence or avirulence phenotypes (Arsenault-Labrecque et al., 2018). In contrast, no sequence polymorphism was detected in *Avr4/6*, indicating that certain *Avr* genes were conserved owing to functional constraints or reduced evolutionary pressure. The contrasting levels of diversity among *Avr* loci suggest that individual effector genes may experience different evolutionary trajectories depending on their interactions with soybean host resistance genes.

Overall, this study revealed substantial pathogenic, genetic, and haplotype diversities among *P. sojae* isolates collected from soybean-growing regions in the Republic of Korea. The extensive diversity observed among South Korean *P. sojae* isolates has important implications for disease management and resistance breeding of soybean. Park et al. (2025) have recently demonstrated that resistance to South Korean *P. sojae* isolates GJ3053 and AD3617 in soybean is controlled by multiple genomic regions and highlighted *Glyma.03g036500* as a promising resistance-related gene. However, rapid evolution of *Avr* genes through sequence diversification and gene loss may reduce the long-term effectiveness of soybean cultivars that rely on a single *Rps* resistance gene. Therefore, continuous monitoring of the pathogen populations and *Avr* gene diversity will be essential for developing durable resistance strategies.

## Conclusion

This study revealed substantial pathogenic, genetic, and haplotype diversity among *P. sojae* isolates collected from soybean-growing regions of South Korea. The predominance of highly virulent isolates, together with the identification of five distinct pathotypes, highlights the widespread occurrence of diverse *P. sojae* populations. Frequent deletions of *Avr3a* and *Avr1a*, along with extensive sequence polymorphisms and high haplotype diversity; indicate that both gene loss and sequence diversification contribute to *Avr* gene evolution and virulence variation. Generally, these findings demonstrate the dynamic nature of *P. sojae* populations in the Republic of Korea and emphasize the importance of continuous monitoring of pathogen diversity to support effective disease management. The extensive pathogenic and molecular diversity observed in this study highlights the ongoing evolution of *Avr* genes within field populations and the potential risk of resistance breakdown. Therefore, deploying soybean cultivars with durable, broad-spectrum resistance, together with pyramiding multiple resistance genes and integrating diverse disease-management strategies, will be essential for achieving sustainable control of PSSR.

## Data Availability

The datasets presented in this study can be found in online repositories. The names of the repository/repositories and accession number(s) can be found in the article/Supplementary material.

## Supplementary material

SUPPLEMENTARY TABLE S2 Haplotype distribution of isolates based on their Avr loci and geographical origin.
